# Bilateral common iliac vein stent migration

**DOI:** 10.1016/j.radcr.2022.08.034

**Published:** 2022-09-15

**Authors:** Andrew Waack, Sarah Jaggernauth, Shikha Sharma

**Affiliations:** a University of Toledo College of Medicine and Life Sciences, 3000 Arlington Ave, Toledo, OH 43614, USA; b Jackson Cardiology Consultants Henry Ford Health, 205 Page Ave, Jackson, MI 49201, USA

**Keywords:** Stent migration, May-Thurner Syndrome, Iliac vein

## Abstract

Venous stent migration to the heart is considered to be a rare complication of a common procedure. Therefore, many physicians do not include this complication in their differential diagnosis. We explain why this complication is likely more common than currently thought and why it should be considered as a potential diagnosis. This case describes migration of bilateral iliac vein stents into the right ventricular outflow tract and right interlobar pulmonary artery. We provide multiple imaging modalities demonstrating the migrated stents. We believe radiologists should be cognizant of this complication and consider it as a potential diagnosis. Hopefully, this will create a greater awareness of this life-threatening complication of venous stent placement.

## Introduction

Venous stents are often placed to maintain or restore vein patency. Migration of these stents through the venous system to the heart is considered to be a rare complication. However, there are many reported cases in the literature of this event [1], [2], [3], [4], [5], [6], [7], [8]. Additionally, many cases are asymptomatic and detected incidentally, implying that additional cases go unrecognized [1]. Lastly, there is no standardized reporting system to document stent migration. Therefore, the true incidence is unknown and likely significantly higher than currently believed [1]. This case report adds to existing literature documenting venous stent migration to the heart. It is of importance so that physicians’ awareness of this potentially lethal complication is heightened because it may not be as rare as perceived.

## Case presentation

**Fig. 1:**
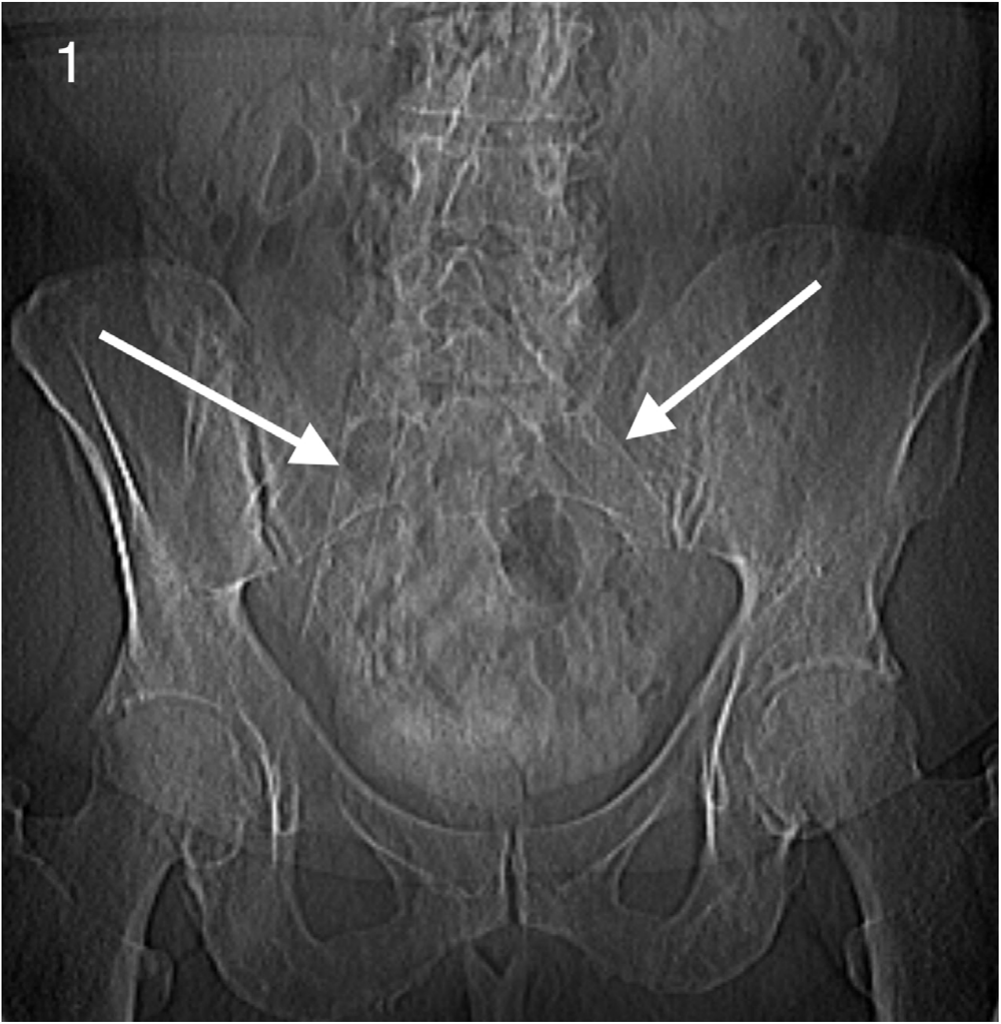
Scout view radiograph demonstrating bilateral common iliac vein stents placed for May-Thurner Syndrome (white arrows).

A 74-year-old female presented to her cardiologist for episodes of atrial flutter and nonsustained ventricular tachycardia. The patient had a dual chamber permanent pacemaker placed 7 weeks prior for complete heart block. Pertinent past medical history included bilateral common iliac vein stent placement 5 years prior for May-Thurner Syndrome (Fig. 1). The patient was on warfarin prophylaxis secondary to prior venous thromboembolism.

The patient reported dyspnea on exertion originating at approximately the same time as the pacemaker placement. On physical exam, the pacemaker scar was well healed with no erythema or tenderness; however, significant jugular vein distension was noted. Respiratory exam was unremarkable. Cardiovascular exam demonstrated normal rate and regular rhythm, although a murmur was heard. Edema was present in both legs. Easy bruising was demonstrated, likely due to warfarin use. There were no significant lab abnormalities. An echocardiogram conducted 7 months prior demonstrated normal chamber sizes, left VEF of 55%-60%, left ventricular hypertrophy, borderline diastolic function, no significant valvular dysfunction and normal pulmonary pressures. A chest radiograph performed 1 week prior to this encounter demonstrated an absence of pulmonary edema and stable lead positioning. Telemetry/EKG on the day of visit showed an AV paced rhythm.

The patient was on atenolol (originally bisoprolol) and diltiazem prior to this office visit; these medications were discontinued during this encounter, and she was started on metoprolol succinate to improve her left ventricular ejection fraction.

**Fig. 2:**
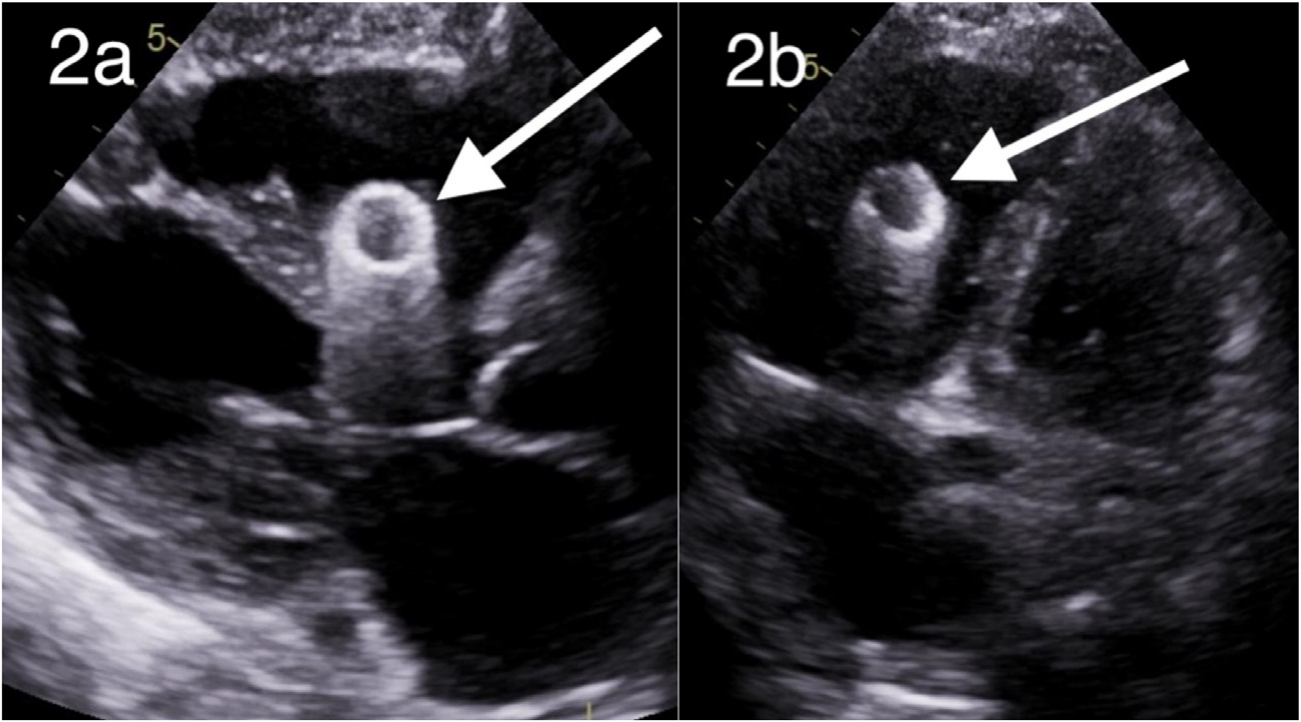
Parasternal long axis view transthoracic echocardiogram demonstrating migrated venous stent in the right ventricle (A, white arrow). Four-chamber view transthoracic echocardiogram demonstrating migrated stent in the right ventricle (B, white arrow).

A transthoracic echocardiogram was performed the following day (Fig. 2). The left ventricle demonstrated mild hypertrophy and mildly depressed systolic function, with an ejection fraction of 40%-45%. The absence of an A wave was consistent with atrial fibrillation/flutter. The atria were mildly dilated as well. There was mild mitral valve regurgitation and trivial regurgitation in the aortic and tricuspid valves. A hyperechoic circular mass was noted close to the right ventricular outflow tract (Fig. 2). The patient was referred to the emergency department (ED). Follow-up CT imaging was also recommended.

The patient presented to the ED following echo findings suggestive of a potential mass in the right ventricle. The patient reported dyspnea of her “normal severity,” fatigue, and weakness. She did not report hemoptysis, pleuritic chest pain or shortness of breath. Cardiovascular exam revealed irregular rhythm, 2+ radial pulses bilaterally, murmur, and intermittent nonsustained runs of ventricular tachycardia with intermittent flutter, which was the patient's baseline. She also exhibited edema in both legs and pale skin. All other physician exam findings were within normal limits. Vitals were as follows: BP: 144/72 mmHg, HR: 94 bpm, RR: 16 rpm, SPO2: 93%.

Although she reported an INR of 3.1 the previous day, her Wells score indicated a moderate risk of pulmonary embolism. She requested to not undergo lab testing, electrocardiogram or admission and expressed a desire to leave. However, a noncontrast enhanced CT was performed to evaluate the foreign body in the right ventricle.

**Fig. 3:**
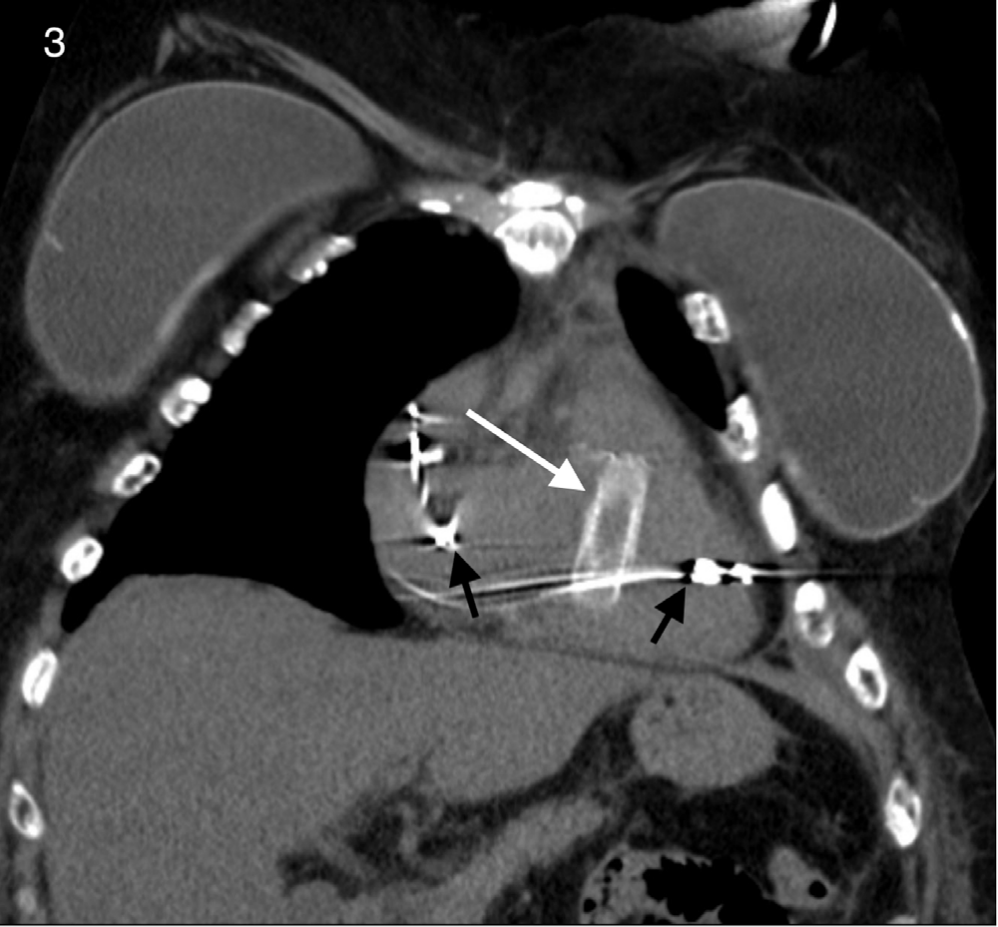
Coronal view CT demonstrating a migrated stent in the right ventricular outflow tract (white arrow). Black arrows indicate pacemaker leads placed for complete heart block.

**Fig. 4:**
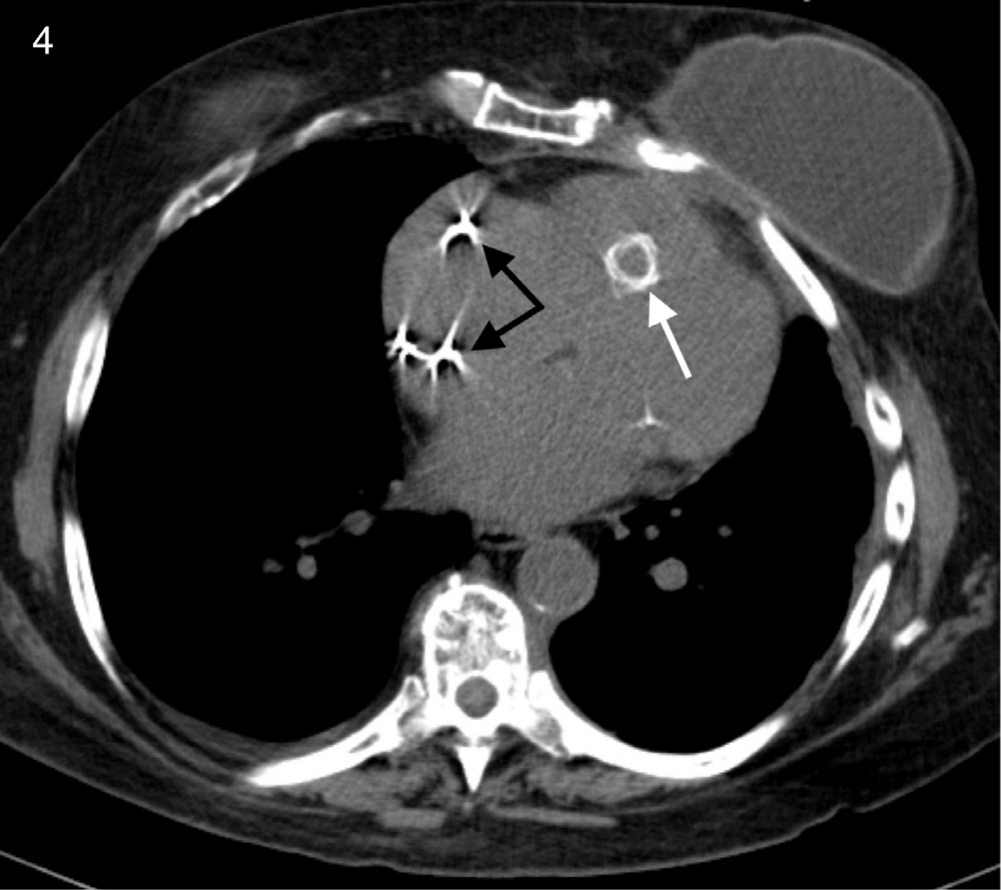
Axial view CT demonstrating a migrated stent in the right ventricular outflow tract (white arrow). Black arrows indicate pacemaker leads placed for complete heart block.

**Fig. 5:**
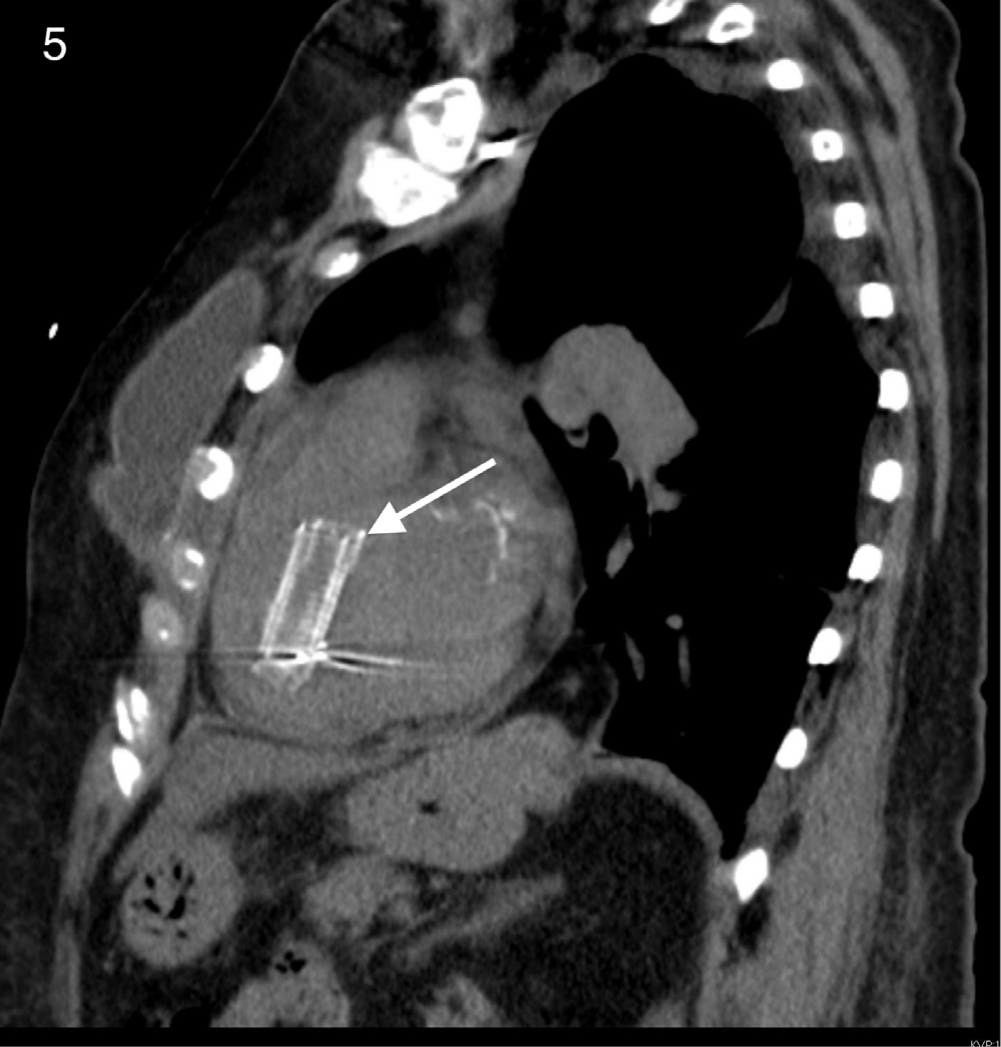
Sagittal view CT demonstrating a migrated stent in the right ventricular outflow tract (white arrow).

**Fig. 6:**
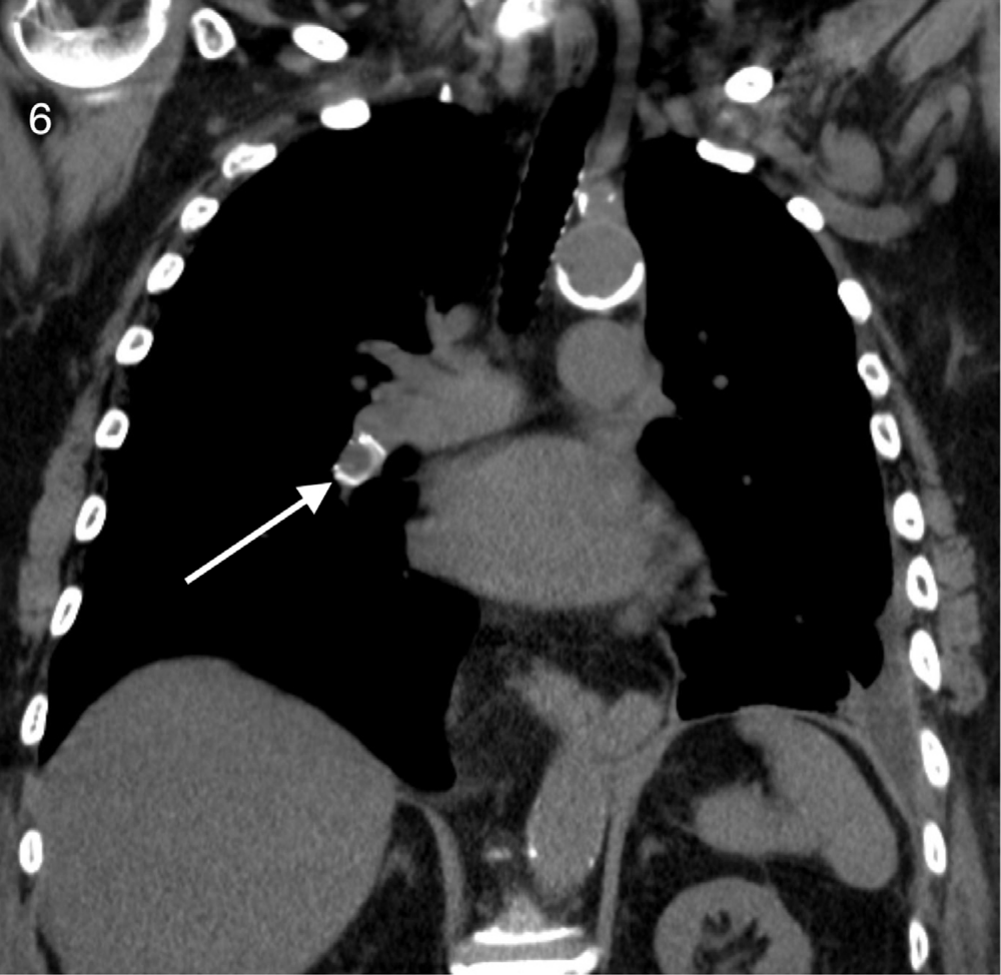
Coronal view CT demonstrating the second migrated stent in the right interlobar pulmonary artery (white arrow).

**Fig. 7:**
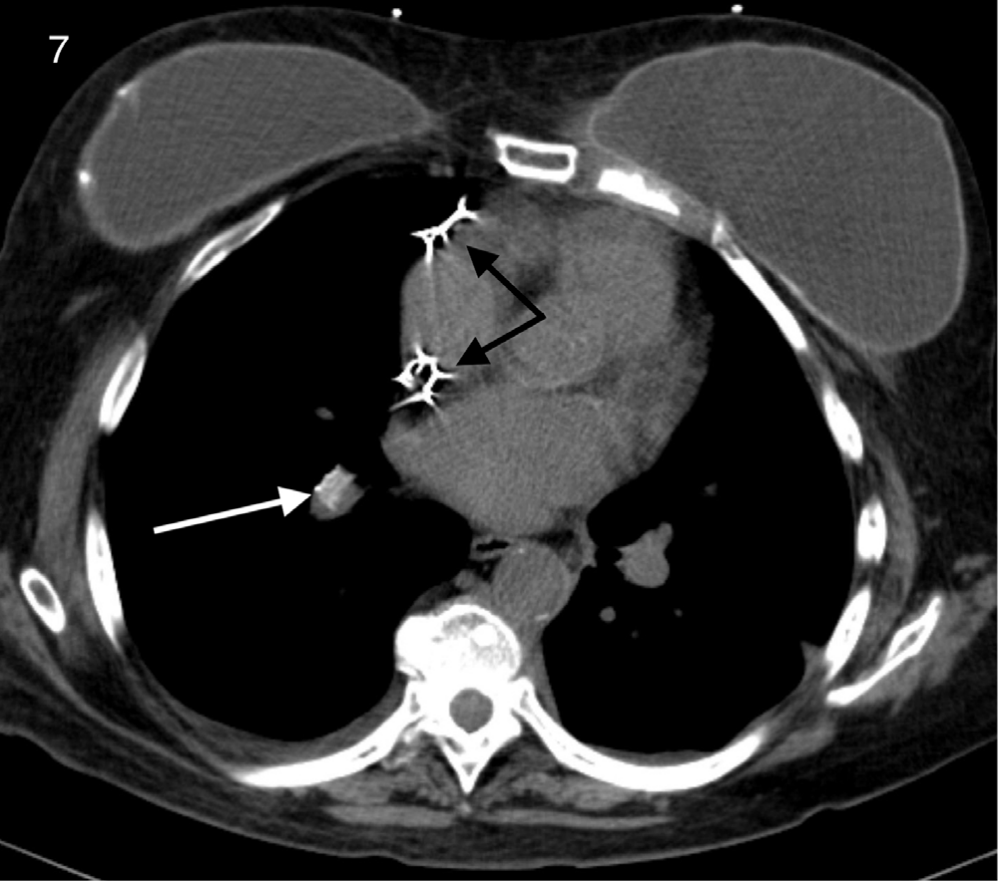
Axial view CT demonstrating the second migrated stent in the right interlobar pulmonary artery (white arrow). Black arrows indicate pacemaker leads placed for complete heart block.

The noncontrast enhanced CT demonstrated migration of both iliac vein stents, one into the right ventricular outflow tract (Fig. 3, Fig. 4, Fig. 5) and the other in the right interlobar pulmonary artery (Figs. 6 and 7).

Following imaging findings of migrated iliac stents, the patient was transferred to a nearby academic medical center for further treatment, as there was no cardiothoracic surgery service available at the primary hospital. The patient later underwent uneventful surgical removal of the ectopic stent in the right ventricle. During surgery, it was noted that the tricuspid valve was severely damaged by the stent and required subsequent replacement. There was also dislodgement of the right atrial lead, which presumably caused the patient's arrhythmia. The pacemaker lead was reattached. It was thought that the risk posed by the removal of the interlobar stent was too great, and it was left in place. The patient has recovered and complains of only mild residual shortness of breath.

## Discussion

Venous stent migration is considered a rare complication. However, there are several documented cases in the literature. Sayed et al. completed a systematic review in 2021 to identify all reported cases of iliofemoral, iliocaval, or thoracic central venous stents that had migrated from their primary source between 1994 and 2020. They identified 54 cases of stent migration in 52 patients placed for a variety of conditions, most commonly central venous obstruction (38.8%) or post-thrombotic syndrome (20.3%) [1]. Additionally, a case report and review of the literature by Mando et al. identified 12 cases (including their reported case) of venous stent migration in patients with May-Thurner Syndrome (MTS), although 2 of these cases were accounted for in Sayed's review, thus only representing an additional 10 cases. Additionally, we were able to identify several cases of venous stent migration that were not included in either above review [3], [4], [5], [6], [7], [8]. In the reported cases, stents were displaced from several primary locations, including the common iliac veins, both vena cava, brachiocephalic veins and subclavian veins, among others [1], [2], [3], [4], [5], [6], [7], [8]. Various stent migration sites have been reported as well, including the right atrium, right ventricle and pulmonary artery [1], [2], [3], [4], [5], [6], [7], [8]. The symptoms of a migrated stent are variable. Notable symptoms of migration include chest pain, dyspnea and arrhythmias [1]. Other patients have also been reported to present with tricuspid valve regurgitation [3]. Also, blood vessels may perforate or thrombose [3]. Interestingly, 41.6% of the cases reviewed by Sayed et al. were asymptomatic and were found incidentally during imaging for comorbidities. Kim describes an incidental finding of a stent migrating into the right ventricle during imaging for a pulmonary embolism. Yet another case describes an incidental finding of a stent displaced into the right ventricle during an echocardiogram prior to a routine saphenous vein ablation [5]. The high prevalence of asymptomatic cases implies that many events of stent migration go unrecognized, thus lowering the reported incidence of this complication.

Several factors are responsible for causing stent dislodgment from their primary site. There are several causes for post-deployment stent migration, including undersizing of the stent for the targeted blood vessel, variations in the diameter of the blood vessel throughout the cardiac and respiratory cycles, inadequate ballooning and excessive shoulder movement [4]. Other cases have reported more rare events causing migration, including thigh massage and trauma [6,7]. Improper stent size is arguably the single most important factor responsible for stent migration. Sayed et al. found that smaller diameter and/or shorter stents migrate more often than larger diameter and/or longer stents, specifically: 82.6% of migrated stents were less than 60 mm in length, and 93.6% were 14 mm or less in diameter. There are several mechanisms by which stents can become dislodged and migrate, and selecting the proper stent size appears to be the most important factor to consider.

There is no defined treatment protocol for managing stent migration. Steinberg et al. outline 3 management strategies for stent migration, including expectant monitoring, endovascular retrieval, or surgical removal. Sayed et al.’s review demonstrates retrieval was attempted in 85.5% of cases, with endovascular techniques utilized in approximately two-thirds of retrieval attempts. Surgical removal of ectopic stents within the heart presents an extreme challenge with many potential life-threatening complications, including arrhythmias, valvular injury, papillary injury, heart perforation and tamponade [3,5]. Four patients who underwent open heart surgery for stent retrieval in Mando's review all suffered significant complications, including stroke, arrhythmia, tamponade, chord rupture, and leaflet damage; consequently, the authors recommend initial attempts at retrieval through endovascular snaring, followed by surgery if endovascular techniques fail.

Venous stent migration is considered a very rare complication. However, it is important for physicians to be aware of this complication because it presents with significant morbidity and mortality, and its true incidence is likely higher than one would believe from the literature.

Steinberg reports a case in which a patient suffering stent migration to the right atrial wall and septum expired in the ED, partially due to a delay in consulting cardiothoracic surgery; the authors state that stent migration was not initially considered in the differential, and by the time migration was suspected and diagnosed, the patient had decompensated and ultimately expired. This tragic case demonstrates both the danger this complication presents and the consequences of being unaware of it, underscoring the need for physicians to be aware of this complication.

Stent migration should be kept in the differential because it is likely more common than reported. There are several factors responsible for artificially deflating the number of reported cases, including lack of awareness of reporting databases, unwillingness to report complications and common asymptomatic presentations. Many stent migration complications go unreported. Sayed et al. anecdotally claim it is relatively common to hear of cases that are never reported. A study analyzing complication rates of endovenous ablation demonstrates that the Manufacturer and User Facility Device Experience Registry (MAUDE) contained only a small fraction of actual complications of the procedure in question [9]. In an invited commentary, a coauthor of this study states that over one third of polled physicians were unaware of the existence of the MAUDE database, and that most physicians reported they would not put in the effort to publish their cases [10]. Next, migrated stents may not demonstrate symptoms that prompt imaging. If these asymptomatic cases are to be diagnosed, it must be as an incidental finding during imaging for a comorbidity. In Sayed et al.’s review, 41.6% of diagnosed stent migration cases were incidentally discovered. If there are no comorbidities that indicate imaging or findings are able to evade incidental detection, asymptomatic cases will likely never be diagnosed. The incidence of venous stent migration is likely higher than it appears in the literature, underscoring the need for physicians to be aware of this complication.

Diagnostic radiologists should be aware of stent migration as a potential complication of venous stent placement, as it is often detected incidentally on imaging and can cause significant morbidity and mortality. Furthermore, radiologists should contribute to the growing body of knowledge of this complication by reporting these adverse events and can potentially help care for these patients through interval imaging to monitor stent location. Recommendations have been made to address the under reporting and under diagnosis of venous stent migration. First, to address the issue of underreporting, Sayed et al. propose the institution of a national venous stent registry to record adverse events, and Labropoulos has expressed support for such a database. Next, stents should be assessed for migration [4]. An intermittent surveillance program could help to identify stent migrations, even if they are asymptomatic. These 2 initiatives could help to identify currently unrecognized cases of venous stent migrations and thus increase the reported rates of migration to their true rates.

## Patient consent

Written consent was obtained directly from the patient.
